# Will Cure Bite of Most Deadly Snakes

**Published:** 1904-12

**Authors:** 


### WILL CURE BITE OF MOST DEADLY SNAKES.

The prevention of death from snakebite has been the object of a number of investigations recently undertaken, and two methods of treatment have been developed with a fair amount of success. One is the injection of a specially prepared antivenim, which acts as an antitoxin, and which has proved useful in many cases. The other is treating the afflicted part with permanganate of potash, which has the advantage that this substance can readily be kept at hand and does not require to be specially prepared and stored like the antivenim. The use of permanganate of potash for snakebites was first suggested in India in 1860 by Sir Joseph Fayrer, and subsequent experiments have demonstrated the effectiveness of the treatment. These experiments, unfortunately, were interrupted by the English antivivisection laws, but recently they have been carried further by means of a device lately invented whereby chloroform can be administered continuously to an animal for a space of time in some cases exceeding twenty-four hours.

In experiments thus undertaken on a number of animals, such as rabbits and cats, it was found that when the puncture caused by the snake's bite or for the introduction of the venom was enlarged by lancet into a small wound, which was then rubbed with a crystal of permanganate of potash, the latter acted as a complete antidote to the poison. Five or six animals injected with cobra poison, one of the deadliest of all reptile venoms, survived after receiving this treatment, and a similar number which had been given daboia poison. A special form of lancet adopted for this purpose was exhibited recently at the Royal Society, London, and is intended for general use. It consists of a sharp steel-pointed blade inserted in a wooden handle which is hollow and contains several crystals of permanganate of potash. As soon as the victim is bitten a piece of cloth is tied firmly above the bite, which is then enlarged with the lancet and rubbed with the crystal.

It is intended that these lancets shall be sold as cheaply as possible at all postoffices in India, just as quinine is sold at present, and it is hoped that the mortality from snakebites may be correspondingly diminished.

In the experiments with the animals it was found that the treatment was equally successful with all kinds of venom, and that the results obtained after five minutes had elapsed between the bite and the injection were as good as when but half a minute intervened. It is believed that the outcome ot this investigation, which in its various phases has occupied nearly forty years, will be productive of widespread benefit.—*Southern Drug Journal.*

